# Supplementary figures and images for: Morphology, ultrastructure and function of the sternal gland in two mason bee species (Osmia bicornis and O. cornuta)
Source: PLoS One. 2025 Oct 17;20(10):e0334876. doi: 10.1371/journal.pone.0334876 (PMC12533917; doi:10.1371/journal.pone.0334876)

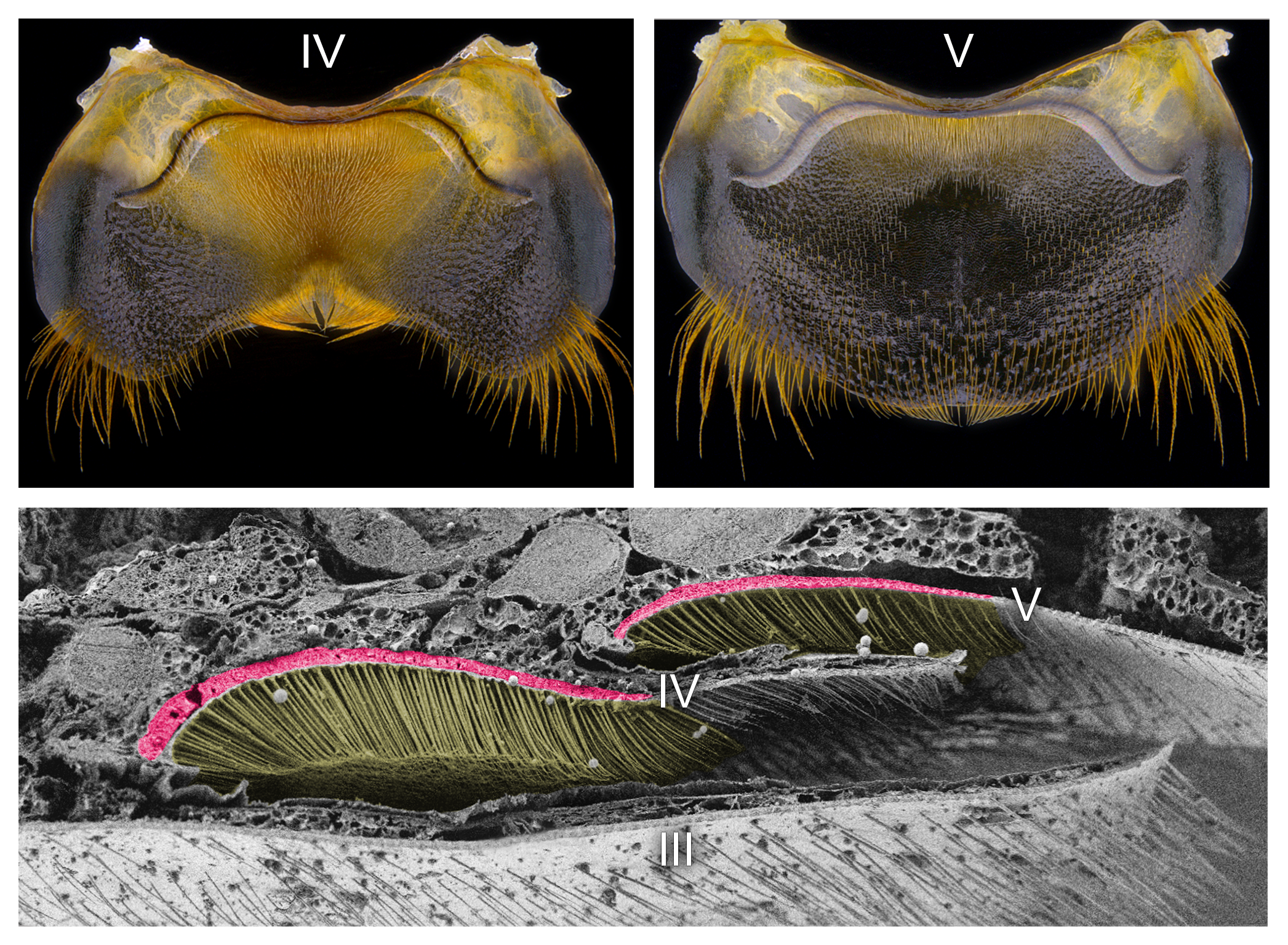

Supplement: S1 — (TIF) [file pone.0334876.s001.tif]
