# Supplementary material for: Morphology, ultrastructure and function of the sternal gland in two mason bee species (Osmia bicornis and O. cornuta)
Source: PLoS One. 2025 Oct 17;20(10):e0334876. doi: 10.1371/journal.pone.0334876 (PMC12533917; doi:10.1371/journal.pone.0334876)
Supplement: Attachment — Submitted filename: Response-to-Reviewers.docx [file pone.0334876.s003.docx]

PONE-D-25-42537

Morphology, ultrastructure and function of the sternal gland in two mason bee species (Osmia bicornis and O. cornuta).

PLOS ONE

**RESPONSE TO REVIEWERS / REVISION TRACK:**

**Reviewer #1:**

However, the structure of the discussion requires change. Authors need to focus on their results and the relationship between these and literature, Latter, they can describe the higher implications of their study. The current version starts with statements about other studies that confuse the goal of their study. A stronmger closing argument root in their results is desirable.

**Response:** We accept the criticism. The discussion was revised to meet your suggestions.

**Line 58.** I suggest using homologous rather than analogous gland morphology as it is unlikely to be a convergence for these congeneric species and in such way the word usage is more rigorous.

**Response:** Thank you for the suggestion. We have reworded the passage.

**Line 79.** Please check typing for “signalling”

**Response:** The manuscript was written in British English throughout. Therefore, we opted for the British spelling.

**Lines 100-102.** It is not clear the role of tissue maceration in the light and TEM microscopy section.

**Response:** We added the reasons for tissue maceration to the sections on both light and electron microscopy.

**Line 136.** Just for precision, authors used plastic petri dishes, right?

**Response:** You are right. We have clarified the statement.

**Please describe** how do you test the effect of manipulation on males, how did you control cross contamination after new tested males are used for the study.

**Response:** Thank you for the comment. We have now included the requested information.

**Line 159.** Please check use of “ore” here

**Response:** The error has been corrected.

**Lines 162.** Please indicate the time frame considered for recording data.

**Response:** Thank you for the comment. This information was provided later in the manuscript. However, it should be stated already at this point. We have added the requested information.

**As a general comment** on morphologic terminology, in Apocrita the region after the constriction is named metasoma, not abdomen, as the propodeum is the first segment of the abdomen that, along the evolution of the group, is now strongly attached to the thorax. In some groups such as ants, this region is named gaster. Abdomen is more appropriate for other insects.

**Response:** You are right, and we are aware of the morphology of the Apocrita. The sternite count was done correctly. Nevertheless, also in bees and wasps the metasoma is named for reasons of simplification and a general understanding also for readers not familiar with the specific morphology of this group usually as “abdomen”. We adopted this general (but incorrect) naming convention. In order to be more precise in our statements, **we changed the terminology** where necessary and **inserted a passage introducing the morphology of the abdomen in Apocrita** for readers not familiar with this group. However, for the ease of understanding we used the term “abdomen” later on, whenever it was used in a correct syntax.

**Lines 192 and 203.** Usually figures are cited in order, thus, figure 2D should be after 2A, and 3A before than 3B.

**Reponse:** Thank you for your comment. We would like to point out that the images in figures 1 to 3 were arranged according to their proportions. However, the labelling of the partial images was inconsistent. **We have changed the labelling**. Throughout, the images are now labelled first order in columns and second order in rows. Furthermore, **we have rearranged the presentation of the results** so that they refer to the figures in the usual order.

**Line 205.** Authors described a basal opening and cite figure 3D, however, the opening is not obvious there.

**Response:** This was a misconception. We were referring to figure 3D with regard to the setae, rather than the openings mentioned in the clause. **We have clarified the statement.**

**Legend figure 4**. Please provide the n of the trials.

**Response:** Thank you for the suggestion. **We have added the number of trials** in all figures.

**Lines 190-197.** The description is detailed but the figures and lettering in them are not very clear. Please improve visibility of the lettering on the EM images

**Response:** You are right. **We have increased the size of the lettering.**

**Line 195.** The lack of consistency in naming sternal glands in social wasps is largely discussed in 30 as this insect group holds several types of secretory cells in the same sternite.

**Response:** We think, you are referring to line 295? **We added the reference**.

**Lines 298-299.** This type of structural statements and a summary of results is desirable at the start of the discussion. It is a bit odd that authors started with comments on other papers rather than their own.

**Response:** We moved the general information on sternal glands to the introduction. **The discussion has been rearranged.**

**Line 305.** Please check the bee name, it is Xylocopa. Finite may be replaced by fine or shallow depressions.

**Response: The spelling of the name has been corrected.**

**Line 312-313.** May the authors explain better why the lack of pores imply absence of accumulation of the secretion?. It is possible that the secreted product permeates the insect wall and accumulate outside?

**Response:** We refer to “secretions” as liquids. In general, that liquids require pores to pass through the cuticle (see the review by Foster and Casas in Annual Review of Entomology, 2025). However, the volatile compounds of the gland secretions may evaporate from the cuticle and accumulate there. We use the term to "volatiles" to indicate this difference. **We have reworded the passage to make the statement more precise**.

**Lines 331-332.** Please check style and grammar, there are several ideas but these appear unconnected or incomplete.

**Response:** We have **reworded the passage**.

**Lines 332, 345.** Osmia should be in italics

**Response:** Thank you. **The formatting has been corrected.**

**The discussion** may benefit of subtitles dealing with the two main topics treated.

**Response: Subtitles have been inserted.**

**Lines 365-367.** Authors seems to suggest that visual cues and individual recognition is possible by the patrolling males. Please check and if so, provide support for this statement.

**Response:** This statement is based on observations made in the course of the behavioural tests. These observations are mentioned and described in various sections of the manuscript. There are no systematic studies quantifying this decline in interest in mate-seeking males. However, as the presentation of a new test male restored the number of approaches, we interpret the observed behaviour as a kind of adaptive short-term “learning” to avoid repeatedly inspecting the same object. **We are not aware of any reference** addressing this behaviour that we could cite.

**Lines 375-376.** Here authors claim that emergence periods do not overlap and thus, chemical differentiation between species is not under pressure to occur, however, in the introduction (lines 78-79), they stated that interspecific signaling is a possibility due sympatry and thus, set an objective to test this idea. They may elaborate a different structure for this topic because the lack of overlapping at emergence is a strong reason, already available to suspect that interspecific signaling is not an issue.

**Response:** You are right. The contradiction arose from managed populations. In nature, the emergence times of the males of both species may overlap. **We have removed the misleading passage.**

**Lines 384-386.** Describing multiple potential explanations without further development or strong evidence is unnecessary and leaves the paper without a significant closing idea. The later is missing in the discussion, what are the most significant implications of the study? Where to go from here?

**Response:** We have restructured the discussion and removed the purely speculative passage at the end. Instead, **we provide a statement on the presence of sternal glands in other mason bees** and we refer to an ongoing project that builds on the present study.
**Reviewer #2:**

Congratulations on a fine study and paper. Results for Osmia bicornis and O. cornuta.

The study of exocrine glands in solitary (non-social) bees are rare. You have produced excellent histological data that convinces me that these sternal glands are real and have the sexual advertisement functions that you claim.

Its fascinating that the carboxylic esters from the sternal gland cells act as an anti-aphrodisiac (i.e. have a deterrent effect) and indicate the sex of the male from a short distance away.

**Line 35.** Yes, but male Osmia have to spend much of their time also finding flowers and drinking nectar to maintain a positive energy balance during their searches for virgin female bees as potential mates, and expending energy during fights with competing males. Yes, scramble competition polygyny as often described by John Alcock.

**Response:** You are right, that males have also to pay their “operating costs”. However, as in many other species, they do not invest in the progeny. There are only some rare cases in Apocirta as a nest guarding to protect it from parasites (described for Tripoxylon) or resource enhancement by defending host flowers against other bees (territoriality in Anthidium). But in mason bees, the fitness of males depend solely on their success in finding (and mating with) virgin females.

**Line 41** should read "pouncing" on any object....

**Response:** Thank you. The error has been corrected.

**Male bees are tuned to the females' CHC bouquet**, which is modified as the females age. You correctly state that newly emerged males can be confused (their cuticular hydrocarbons) with females. These males begin releasing large amounts of species-specific carboxylic acid esters within 3 days of emergence from their cocoons.

**Response:** We are convinced that the CHC bouquet is used in many solitary bees to identify mates. Since in many species’ females are receptive immediately after their emergence from the maternal nest, this nuptial CHC bouquet bears the potential for mistaken identities. Also, males of other species of the tribe Osmiini possess sternal glands and release volatiles. The pattern found in the two mason bees studied here might be a widespread phenomenon.

**Your methods for fixation** and histological prep. and examination are excellent. Ditto for SEM methods.

**Response:**

**Lines 127 - 130.** Please give me a bit more information about how males were presented to other males. These were tethered by a thin thread (made of ____?) at the petiole? You also made behavioral observations in the field as well as the laboratory, and these results agree.

**Response:**

Line 138. How were pentane extracts of sternal glands applied to male bees? How many microliters per bee and how long did you continue observations following topical pentane applications? That males were only tested once and then released is a good regimen.

**Response:** Thank you for the suggestion. **We have added the requested information** and have expanded the methods section to describe the behavioural assay in more detail.

**Line 145.** I see a mention of 5 minutes.

**Response:** Thank you for the tip. The information on observation time was provided to late in the manuscript. **We have added the indications of the observation time** in the description of the tests.

**Your histological descriptions** of the sternal glands, and associated photos, are excellent.

**Histology of sternal gland cells**. That the surfaces of these sternites are not more modified (just dented and with setae) is interesting.

**Response:** Yes, this surprised us. We have had expected to find pores in the cuticle to facilitate the release of the gland secretions.

**I have no issues with your statistical measures** of the SSB approaches etc, or stats in general. I see no reason for a statistician to further evaluate this paper. Use of ANOVA repeated measures test makes sense to me.

**Response:** We are really pleased.

**Figures 4, 5, and 6** are excellent. They both clearly show the results. I see no reason to make changes in these three figures.

**Response:** **We have had to add the number of trials** in all figures

**Your histological results will likely** lead other researchers to look for similar "hidden" sternal glands in other bees in the Megachilidae and elsewhere. There must be similar glands in other bees that we are not aware of, and yet are vitally important for an understanding of their courtship and copulatory behaviors.

**Response:** You are right. In the tribe Osmiini males of all checked species (on the base of a random sample) seem to have a sternal gland. **We included a statement at the end of the discussion.**

**Line 308.** You correctly mention how different the sternal glands in your Osmia bees are from the sternal glands of other insects (e.g. termites, ants, Polistes wasps).

**Response:** This passage was **moved to the introduction** to provide a better background of the study and to concentrate on the results in the discussion.

**The mechanism of "pheromone" release** in Osmia remains unclear. I agree that it is likely a function of the uncontrolled, slow, and continuous release of these secretions. Perhaps discussions with a biophysicist interested in fluid dynamics would be appropriate, but not necessary for the publication of this article.

**Response:** Thank you for the suggestion. **We thought about that** but were not able to identify a colleague that could help with this problem.

**Lines 329 to 335.** Yes, you correctly state that some dorsal mesosomal glands in Xylocopa bees are lined with many thousands of short still setae projecting into the lumen of their male glands. I have been part of such studies in Arizona. We do not fully understand the role of these setae in the release of pheromones from these massive glands. I am not sure that the role of setae for chemical dispersion in Xylocopa vs. Osmia are the same. I could be wrong.

**Response:** The enlargement of the surface is a wide spread pattern in glands releasing pheromones. Setae are an excellent tool reach that. In the published results on the thoracic glands in Xylocopa we were not able to identify pores to release secretions directly. In one case, a pore at the tip of the setae was suggested, but according to our experience (and suggestions from colleagues familiar wis SEM), this could be an artefact due to surface charge. **We agree, that the found similarities are no proof for identical roles of setae** for chemical dispersion, but this assumption seems obvious.

Perhaps as you state, the Osmia chemical dispersion into the boundary layer air and beyond can be aided in the form of discrete puffs when the abdomen is ventilated to bring oxygen into the tracheae.

**Response:** We would like to prove this hypothesis by chemical measurements. But up to now we have no idea how to do that.

**Line 378.** I like the description of these anti-aphrodisiac chemicals as abstinons. A great word.

**Response:** This term was suggested by Schlein et al. in 1981.

**Olfactory flags.** Male bees avoid vain captures and contacts. They do not waste time and energy by courting competitors.

**Response:** This concept was developed in an earlier study. Here we have studied whether the sternal gland secretion, mainly the carboxylic esters, serve the suggested function.

**One potential issue.** I don't seem to have found the figure captions for Figures 1 through 6. I'm sure they are fine, I just don't see them.

**Response:** The figure **captures were included in the text** at the positions, where the figures should appear. You can find them in line 184 ff, 213 ff, 219 ff, 242 ff, 272 ff, and 284 ff of the submitted version of the manuscript.
